# Supplementary material for: Defactinib in Combination with Mitotane Can Be an Effective Treatment in Human Adrenocortical Carcinoma
Source: Int J Mol Sci. 2025 Jul 7;26(13):6539. doi: 10.3390/ijms26136539 (PMC12249900; doi:10.3390/ijms26136539)
Supplement: Supplementary file 1 [file ijms-26-06539-s001.zip › ST1_In silico datasets.pdf]

Supplementary Table S1. In silico datasets used for this study.

ACC: adrenocortical carcinoma; DMSO: dimethyl sulfoxide; n.r.: not relevant

Analysis	Study	Sample type	Treatment	Sample number
Gene expression analysis	GSE140818	Mitotane sensitive HAC15 cell line	50 uM mitotane (18h)	6
		Mitotane sensitive HAC15 cell line	DMSO	6
		Mitotane resistant HAC15 cell line	50 uM mitotane (18h)	6
		Mitotane resistant HAC15 cell line	DMSO	6
	GSE73417	Xenografted H295R cell line	200 mg/kg mitotane (28 days)	4
		Xenografted H295R cell line	Corn oil	4
	GSE143383	ACC	n.r.	43
		Normal adrenal	n.r.	5
	GSE90713	ACC	n.r.	57
		Normal adrenal	n.r.	5
	GSE33371	ACC	n.r.	33
		Normal adrenal	n.r.	10
	GSE12368	ACC	n.r.	12
		Normal adrenal	n.r.	6
	GSE14922	ACC	n.r.	4
		Normal adrenal	n.r.	4
Survival analysis	TCGA-ACC	ACC	n.r.	79
Discrimination analysis	GTEx	Normal adrenal	n.r.	128
