# Supplementary material for: Defactinib in Combination with Mitotane Can Be an Effective Treatment in Human Adrenocortical Carcinoma
Source: Int J Mol Sci. 2025 Jul 7;26(13):6539. doi: 10.3390/ijms26136539 (PMC12249900; doi:10.3390/ijms26136539)
Supplement: Supplementary file 1 [file ijms-26-06539-s001.zip › Supplementary Methods_ext_jav.pdf]

Supplementary Methods

In vitro 2D and 3D ACC models - matrigel-scaffolding and bioprinting

H295R and SW13 adrenocortical cell lines were obtained from LGC Standards GmbH (Wesel, Germany) in the frame of the LGC-ATCC partnership. Culturing and matrigel-scaffolding were done exactly as previously described [50]. Briefly, H295R cells were grown in Dulbecco's Modified Eagle Medium (10–013-CV, DMEM with 4.5 g/L glucose, L-glutamine, sodium pyruvate, Corning, Corning, NY, USA) supplemented with 10% fetal bovine serum (35–079-CV, FBS, Corning, Corning, NY, USA), 2.5% Nu serum (CB-51000, BD Biosciences, Franklin Lakes, NJ, USA), 1% ITS Premix universal Culture Supplement (354,350, Corning, Corning, NY, USA) and 1% penicillin/streptomycin (10,378,016, Thermo Fisher Scientific, Waltham, MA, USA). SW13 cells were cultivated in Leibovitz L-15 Medium (L1518 Sigma, St. Louis, MI, USA) with 10% fetal bovine serum (P30-19375, PAN Biotech, Aidenbach, Germany) and 1% penicillin/streptomycin (10,378,016, Thermo Fisher Scientific, Waltham, MA, USA).

For the 3D matrigel-scaffolded model, Matrigel Matrix (354262, Corning, Corning, NY, United States) was applied as we previously described [50]. Briefly, 250 µl matrigel with 250 µl base media was mixed, and then each well of a 6-well plate was coated with 500 µl diluted matrigel mixture. The matrigel matrix was then polymerized at 37°C for half an hour. Then $1x10^6$ cells/well in 2 ml of complete medium was layered on top of the matrigel that allowed cells to form 3D spheroid structures surrounded by extracellular matrix. Cell Recovery Solution (354253; Corning, Corning, NY, United States) was used to safely recover the cells, cultured on matrigel for additional *in vitro* functional assays, following the manufacturer's instructions.

Imaging of 2D and 3D matrigel-scaffolded models was done by Canon Power Shot A590 IS software using ×50 objective and ×10 ocular.

For 3D bioprinting, two types of bioinks were used: a. cell-containing-gel: 3% alginate and 1% gelatine (Merck-Sigma-Aldrich, Darmstadt, Germany) bioinks were mixed with cells (1 × 107/ml) immediately before printing; b. cell-free-gel, more rigid gel, 6% alginate, and 11% methylcellulose (Merck-Sigma-Aldrich). The scaffold layout (6 layers alternately) was designed with GeSiM Robotics

software and performed by an extrusion-based bioprinter (Bioscaffolder 3.2, GeSiM, Radeberg, Germany). The printing conditions were the following: radius and height (2.5–5 mm, 0.5–1 mm); interlayer angle (90°); the distance of infill (1.5 µm); printing speed (10 mm/s); needle diameter and height (110 µm for cell-free-gel and 50–50 µm for cell-gel); pressure (400 kPa for cell-free-gel and 20 kPa for cell-gel). The scaffolds were post-processed by CaCl2 crosslinking (200 mM, 2 min) and washed twice then maintained in culture media [51-52].

In vitro treatments and functional assays

For *in vitro* treatments mitotane (SML1885, Merck-Sigma-Aldrich, Darmstadt, Germany) at 5 µM and defactinib (ab254452, Abcam, Cambridge, UK) at 1 µM and 5 µM diluted in dimethyl sulfoxide (DMSO, D4540, Merck-Sigma-Aldrich, Darmstadt, Germany) at a final 0.01 V/V% were used.

For determining cell viability and proliferation conventional Alamar Blue assay (DAL1025, Invitrogen, Thermo Fisher Scientific, Grand Island, NY, USA), Sulforhodamine B (SRB) assays, and Trypan Blue assays were used as previously described [50-51]. Alamar Blue, as a redox indicator was used to assess metabolic activity of living cells to quantitatively measure viability as it is recommended by the manufacturer. Fluorescent signals with excitation at 560 nm and emission at 590 nm were detected using a flash spectral scanning multimode reader (5250040, Varioskan, Thermo Fisher Scientific, Waltham, MA, USA) with SkanIt Software 2.4.5 RE.

Before SRB assaying of 3D scaffolds, 10% trichloroacetic acid (60 min; 4°C) fixation, washing steps, and overnight drying were applied. 0.4 m/V % Sulforhodamine B (SRB, S1402, Merck-Sigma-Aldrich, Burlington, MA, USA) was diluted in 1% acetic acid as was added to cells as 50 µl/well for 15 min for 2D and 1 h for 3D cultures at room temperature. The bound SRB was re-dissolved in 10 mM (150 µl/well) Tris base solution (648310-M, Merck-Sigma-Aldrich, Burlington, MA, USA) and measured by LabSystems Multiskan RC/MS/EX Microplate Reader (570 nm; Labsystems International; Transmit Software Version 4.5—Vantaa, Finland). Relative cell proliferation was calculated in the percentage of control cells

For both Alamar Blue and SRB assays, 3D scaffolds were transferred into new 96-well plates (1 scaffold/well/100 µl media) directly before the measurements.

For cell number and the ratio of dead cells were assessed by 0.4% Trypan Blue staining (15250061, Gibco, Thermo Fisher Scientific, Waltham, MA, USA). For analyzing 3D models after spheroid formation, the cells were trypsinized then stained and analysed [50]. All measurements were done at least three times (biological replicates) with one to three technical replicates in each.

Xenograft model and dose-testing with non-lethal outcomes

The H295R xenograft model was established as we previously described [17]. Briefly, 1×10^6 H295R cells mixed in a 1:1 ratio with matrigel (356237, Corning, Corning, NY, United States) with a final volume of 200 µl were injected subcutaneously on the flanks of 6–8-week-old male SCID mice. After tumour formation, in the first experiment 200 mg/kg/day mitotane treatment (n=8) or corn oil (as vehicle control, n=8) was administered *per os* (gavage) in a final 80 µl for 5 weeks.

Prior to the combined treatment, a three-week *in vivo* dose-testing toxicology study was performed on three SCID mice. In combined treatment 200 mg/kg/day mitotane in 40 µl corn oil was mixed with 50 mg/kg/day defactinib in 40 µl corn oil with 5% DMSO and administered to the animals by *per os* gavage. To investigate toxic effects and the tolerance of the combination therapy we determined the animal weight, behavioural changes and alterations in appearance according to the general distress scoring sheet [16]. No weight loss or other toxic signs were observed.

In the second xenograft experiment treatment groups were designed as: i) 200 mg/kg/day mitotane in 80 µl corn oil (n=8), ii) 50 mg/kg/day defactinib in 80 µl corn oil with 5% DMSO (n=9), iii) 200 mg/kg/day mitotane in 40 µl corn oil with 50 mg/kg/day defactinib in 40 µl corn oil with 5% DMSO (n=9) and iiii) 80 µl corn oil (n=9) control for 6 weeks.

In both experiments, tumour sizes were measured by calliper and tumour volume was calculated as $\text{width}^2 \times \text{length} \times 0.5$.

In vivo experiments and dose-testing with non-lethal outcomes were authorized by the National Council for Scientific Ethics in Animal Experiments PE/EA/801-7/2020; PEI/001/1738-3/2015; PE/EA/1461-7/2020.

In accordance with the approved protocol, in the xenograft experiments, tumour sizes remained below 1.5 cm in diameter, with no signs of ulceration, bleeding, or necrosis. Additionally, the animals

showed no indicators of distress, pain, or discomfort, such as significant weight loss, reduced mobility, or difficulty accessing food and water. The tumours did not interfere with the animals' normal physiological functions.

Steroid Hormone Measurements by HPLC-MS/MS

Cortisol and cortisone hormone measurements of cell culture media and mouse sera were performed by HPLC-MS/MS. We used our previously published and applied protocols [50; 59]. Briefly, reference materials (cortisol solution 1 mg/ml dissolved in methanol and cortisone 250 mg) and the internal standard (certified reference material: 9,11,12,12-D4-cortisol 100 µg/ml solution, dissolved in methanol) were purchased from Sigma-Aldrich (Burlington, MA, USA). LC-MS grade water, LC-MS grade methanol, and LC-MS grade formic acid were purchased from VWR International (Radnor, PA, USA).

For sample preparation 10 µl internal standard (2.76 µmol/l) was added to 90 µl cell culture media. Protein precipitation was carried out by adding 300 µl acetonitrile. After vortexing, samples were centrifuged for 5 minutes at 13,500 rpm. The supernatant was diluted in 1:1 proportion with LC-MS grade water after which the sample was ready for analysis.

LC-MS/MS assays were performed on a Perkin-Elmer Flexar FX10 ultra-performance liquid chromatograph coupled with a Sciex 5500 QTRAP mass spectrometer. For chromatographic separation, a Phenomenex Kinetex C18 stationary phase column (50 × 2.1 mm, 1.7 µm) attached to a Phenomenex Security Guard Ultra C18 guard column (2 × 4.6 mm) was used (Gen-Lab Ltd., Budapest, Hungary). The mobile phase consisted of water containing 0.1% (v/v) formic acid (A) and methanol containing 0.1% (v/v) formic acid (B). The gradient program and mass spectrometry assay were run as we previously described [50; 59].

Nuclear magnetic resonance (NMR) spectroscopy

NMR measurements were conducted on a 600 MHz Varian NMR SYSTEM spectrometer at 298 K. The ^{1}H NMR spectra were referenced to the residual solvent signal of DMSO-d_6. Chemical shifts (δ) and coupling constants (J) are given in ppm and Hz, respectively.

Defactinib:^{1}H NMR (600 MHz, DMSO-d_6) δ 9.82 (s, 1H), 8.68 (d, *J* = 2.5 Hz, 1H), 8.58 (d, *J* = 2.5 Hz, 1H), 8.31 (s, 1H), 8.18 (d, J = 4.8 Hz, 1H), 7.67–7.59 (m, 4H), 7.40 (t, J = 5.3 Hz, 1H), 5.00 (d, J = 5.1 Hz, 2H), 3.22 (s, 3H), 3.20 (s, 3H), 2.75 (d, J = 4.5 Hz, 3H).

Mitotane: 1H NMR (600 MHz, DMSO-d6) δ 7.84 (dd, J = 7.9, 1.6 Hz, 1H), 7.53–7.49 (m, 2H), 7.45 (dd, J = 8.0, 1.3 Hz, 1H), 7.43–7.40 (m, 2H), 7.40–7.36 (m, 2H), 7.29 (td, J = 7.7, 1.6 Hz, 1H), 5.07 (d, J = 10.4 Hz, 1H).

Transcriptome sequencing and bioinformatics

Total RNA was extracted with Qiagen MiRNeasy Mini kit (217004, Qiagen, Hilden, Germany). following the manufacturer's instructions. Nucleic acid purity and quantity were analysed with NanoDrop 1000 Spectrophotometer (Thermo Fisher Scientific, Waltham, MA, USA.), and RNA integrity was tested by Bioanalyzer Bioanalyzer RNA 6000 Nano assay.

Library preparation and sequencing were done as previously described [55]. Briefly, polyA NGS library preparation was completed using NEBNext Ultra II Directional RNA Library Prep Kit for Illumina with Purification Beads (NEB #E7760S/L) strictly following the manufacturer's instructions. Sequencing was run on the Illumina Novaseq platform (NovaSeq 6000 SP 300cycles (2x150 bp)) with data output 100M PE reads/sample. Fastq file processing was done using the R package.

Sequenced RNA-seq data were mapped to the human genome (hg38) obtained from Gencode using the v44 annotation. Reads were mapped using the *STAR*aligner [60] version 2.7.11a, applying previously published benchmarked settings [61]. Raw read quantification was obtained by *FeatureCounts*version 2.0.2 [62]. Differential expression analysis was performed using *DESeq2* [63 version 1.38.3, while TMM-FPKM normalized expression was calculated using *edgeR* [64] version 3.40.2 in R (v4.2.1). Expression heatmap was plotted using the *ComplexHeatmap* [65] package (version 2.14.0) in R.

Principal component analysis was performed using iDEP.91 (http://bioinformatics.sdstate.edu/idep/). For pathway and gene ontology analysis gene set enrichment was performed using ToPPGene Suite (https://toppgene.cchmc.org/). Raw data will be uploaded to NCBI upon manuscript acceptance.

In silico datasets and genomic characterization

For the assessment of gene expression alteration in normal adrenal gland, adrenocortical carcinomas, mitotane response, and resistance *in silico* datasets were obtained from NCBI Gene Expression Omnibus (**Supplementary Table ST1**). Data were re-analyzed using the GEO2R algorithm to avoid biases originating from different analysis settings and the usage of different software. For identification of significant ($p<0.05$) gene expression changes Benjamini & Hochberg (false discovery rate) adjustment was applied. For genomic characterization (copy number analysis, DNA methylome, gene and protein array expression profile analysis), genomic data of 92 human adrenocortical cancer samples were retrieved from The Cancer Genome Atlas through cBio Portal (https://www.cbioportal.org/; accessed on July 25, 2024). ssGSEA using the ssgsea function of the corto(version 1.2.2) [57] package in R (version 4.2.1) was used for gene signature correlations to prevent results from being skewed by highly abundant genes. Based on the FAK signaling gene signature principal component analysis (PCA) was also used to discriminate normal adrenal gland and adrenocortical carcinoma samples [58].

Statistical analysis

Based on the sample distribution determined by the Shapiro-Wilks test and group number, unpaired T-test, Mann-Whitney U test, or one-way ANOVA (with Tukey's multiple comparison test) was used to identify statistical significance among different groups with FDR adjustment for multiple testing correction. Genes correlating with *PTK2* were revealed by Spearman's correlation. Kaplan-Meier analysis assessed the effect of disease-free and overall survival using the median as the cut-off. For comparison of survival curves and the estimates of the hazard ratios Cox proportional-hazards model.

Supplementary Figure legends

Supplementary Figure S1. Characterisation of different ACC models. (**A**) Viability of 2D monolayer and 3D matrigel-scaffolded model during 14 days. (**B**) 5 and 15 uM mitotane effect of cell viability/cell growth in monolayer cultures. (**C-D**) proliferation, (**E-F**) dead cell ratio and (**G-H**) cortisol production of H295R cells cultured in 2D and in Matrigel-scaffolded 3D environment.

Supplementary Figure S2. FAK signalling members in human ACC tumour tissues. (**A**) Association of FAK encoding *PTK2* expression with the copy-number alterations. (**B**) Association of FAK encoding *PTK2* expression with the methylation. Effect of members of FAK signalling on overall survival: (**C**) ECM components of FAK signaling, (**D**) cytoplasmic membrane components of FAK signaling and (**E**) downstream FAK signalling members and endpoints.

Supplementary Figure S3. The effect of FAK signature on ACC patient survival. FAK signature represents FAK activity characterized with 11 genes (*PTK2, CTNNB1, BIRC2, COL1A1, COL4A2, CCND1, ITGA1, ITGA8, ITGA9, RELN, IGF1R*) using ssGSEA. Patients with high FAK signature has worse progression-free (**A**) and overall survival (**B**) in univariate and multivariate analyses as well. (**C**) FAK signature discriminated ACC tissues from the normal adrenal gland in principal component analysis

Supplementary Figure S4. (**A**) Effect of defactinib at different concentrations on H295R cell viability. (**B**) Defactinib and mitotane have no major visible morphological effect on 2D monolayer H295R ACC cells. Defactinib has no significant effect on cortisol production of H295R ACC cells in either 2D monolayer (**C**) or 3D matirgel-scaffolded model (**D**).
(**E**) Effect of defactinib and mitotane on H295R cell migration.

Supplementary Figure S5. (**A**) Chemical structures of defactinib and mitotane. (**B**) Full ^{1}H NMR spectra of defactinib, mitotane and mitotane-defactinib mixture. The ^{1}H NMR analysis demonstrated no chemical reaction between defactinib and mitotane; and both drugs retained their structural integrity in DMSO-d_6.

Supplementary Figure S6. H295R xenograft tumour weight (**A**) and mice serum cortisol level (**B**) and tumour Ki67 indices (**D**) upon mitotane, deactinib and combined treatment. (**C**) Representative image of Ki67 staining with positive and negative cell muclei. Representative microscopic histology images of lung (**E**) and retroperitoneal (**F**) metastases.
