# Supplementary figures and images for: Defactinib in Combination with Mitotane Can Be an Effective Treatment in Human Adrenocortical Carcinoma
Source: Int J Mol Sci. 2025 Jul 7;26(13):6539. doi: 10.3390/ijms26136539 (PMC12249900; doi:10.3390/ijms26136539)

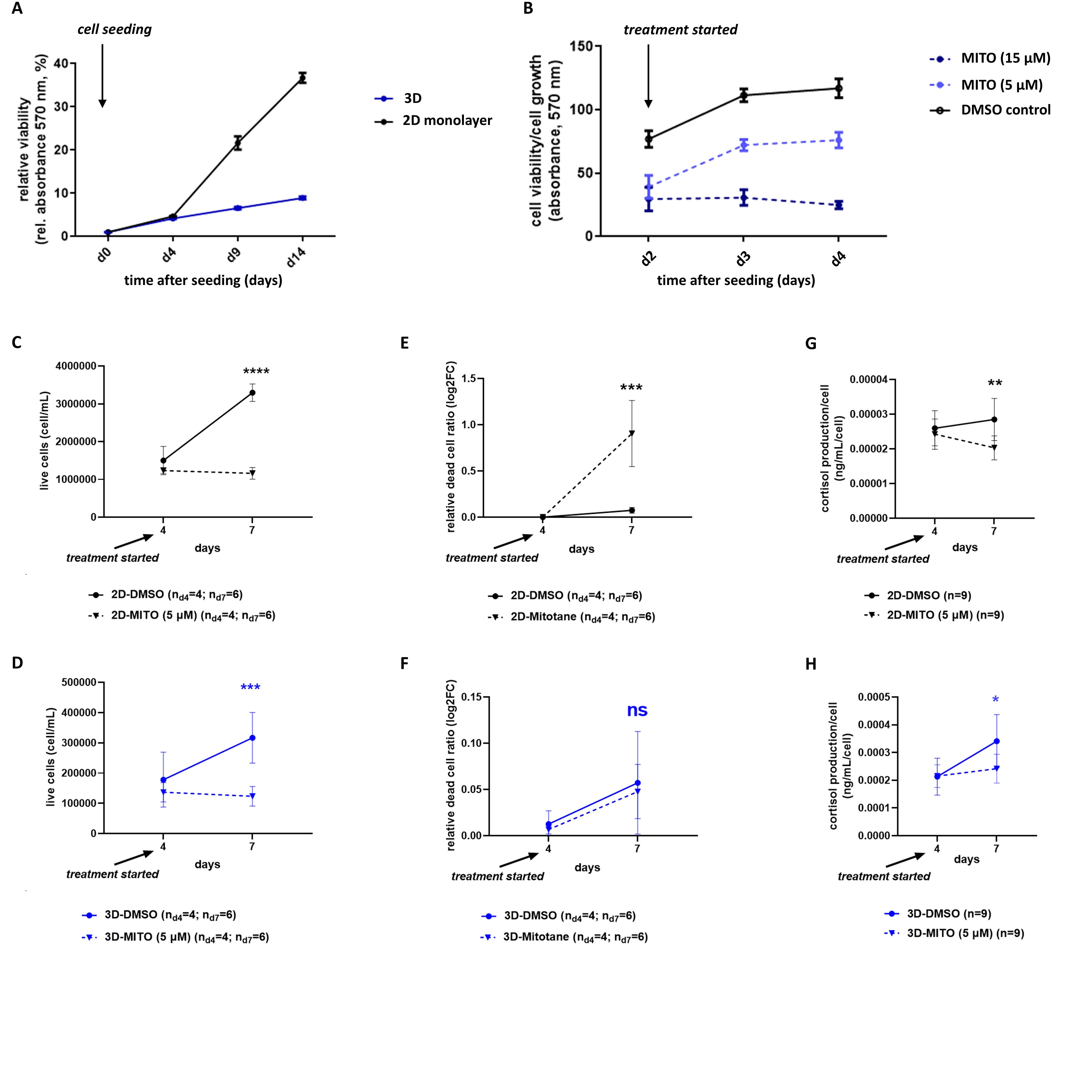

Supplement: Supplementary file 1 [file ijms-26-06539-s001.zip › SF1_1000.jpg]

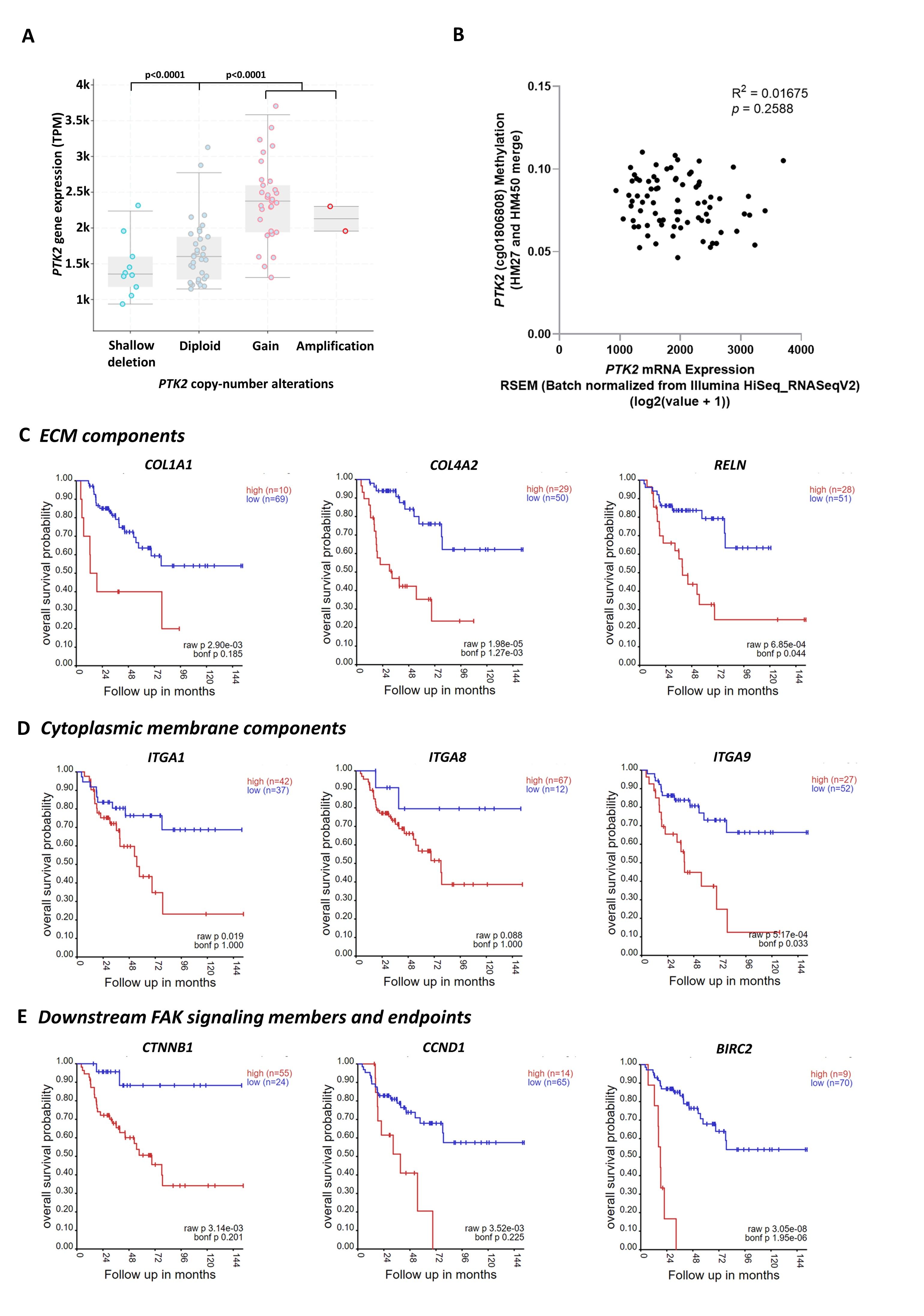

Supplement: Supplementary file 1 [file ijms-26-06539-s001.zip › SF2_1000.jpg]

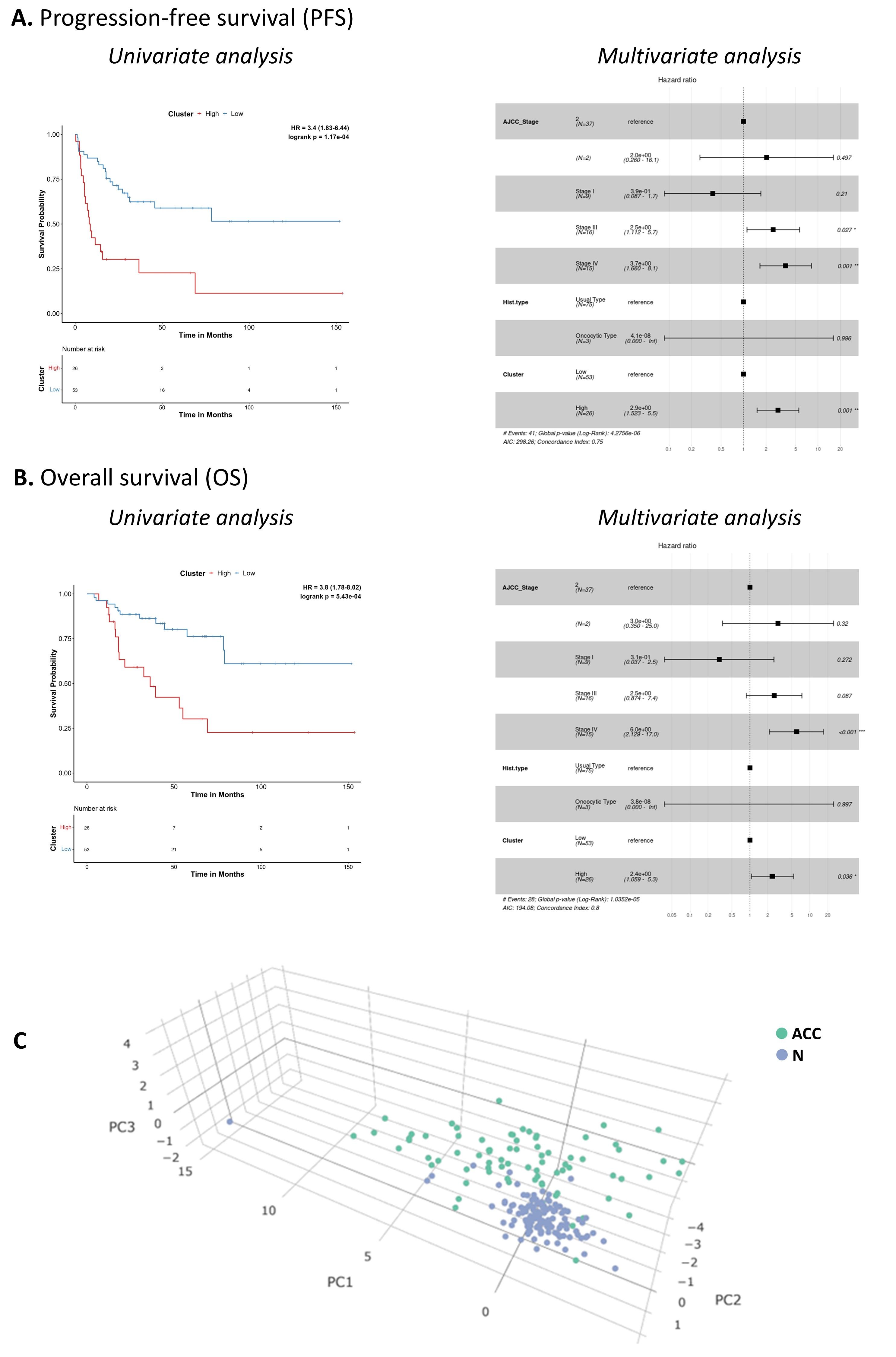

Supplement: Supplementary file 1 [file ijms-26-06539-s001.zip › SF3_1000.jpg]

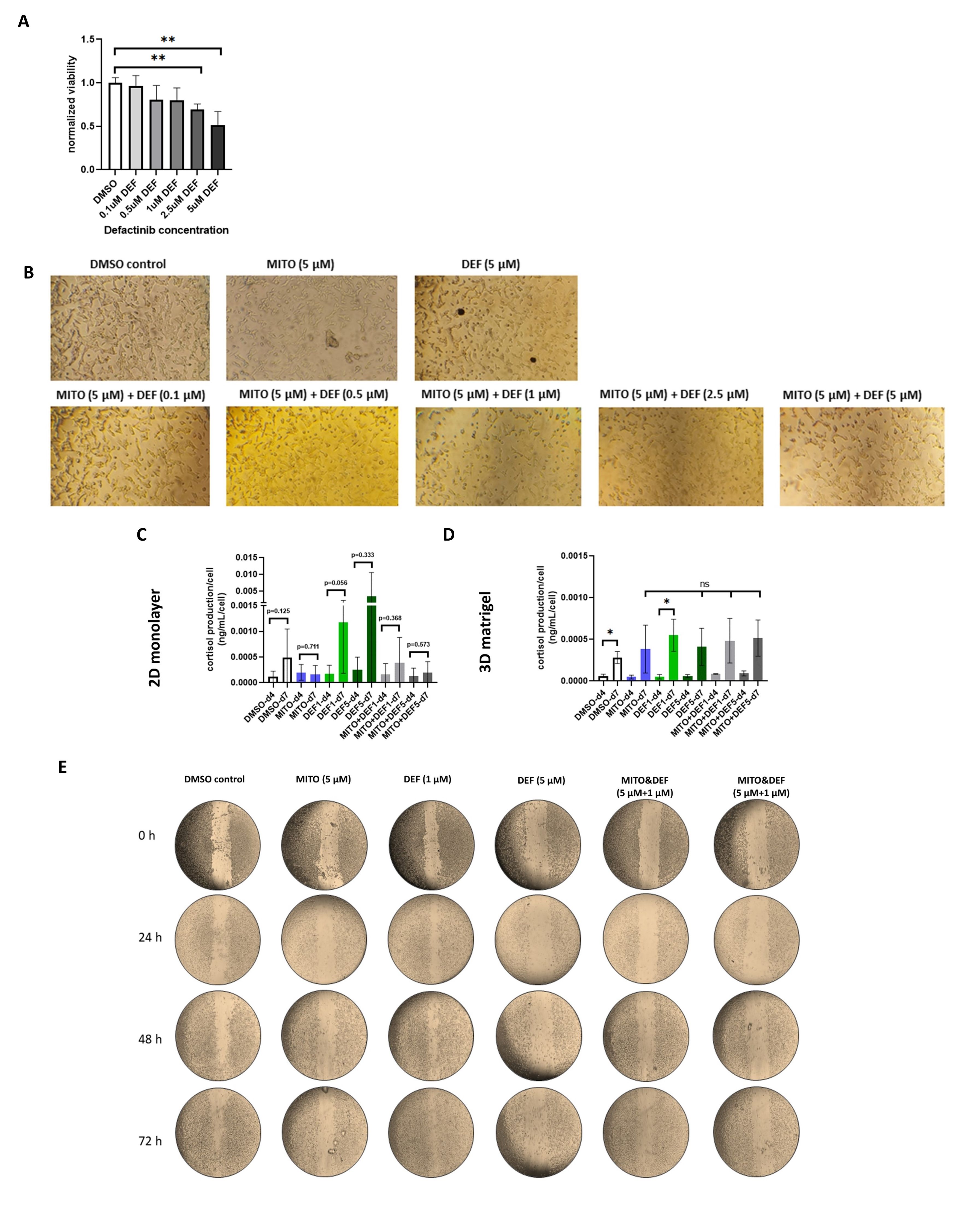

Supplement: Supplementary file 1 [file ijms-26-06539-s001.zip › SF4-镴.jpg]

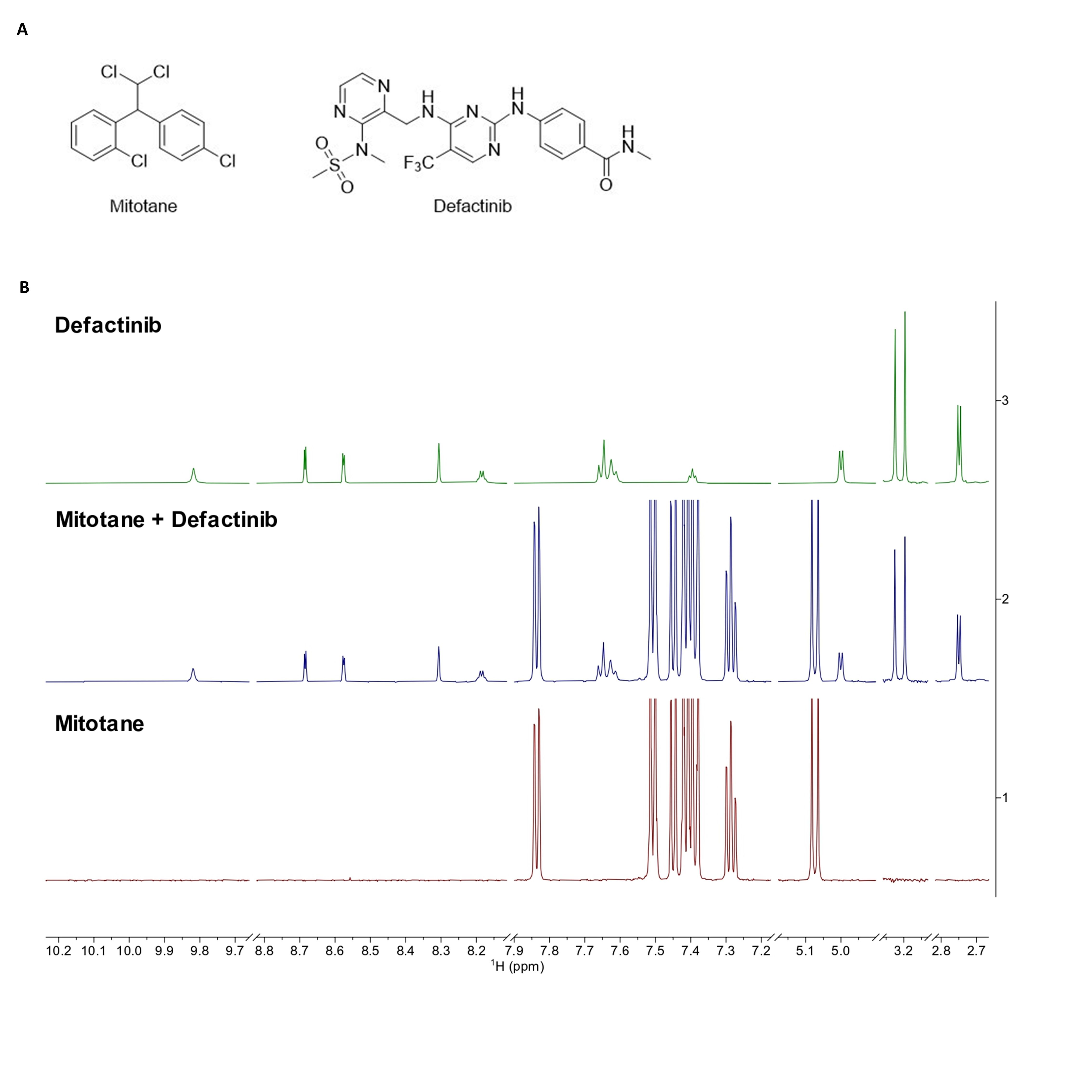

Supplement: Supplementary file 1 [file ijms-26-06539-s001.zip › SF5_1000.jpg]

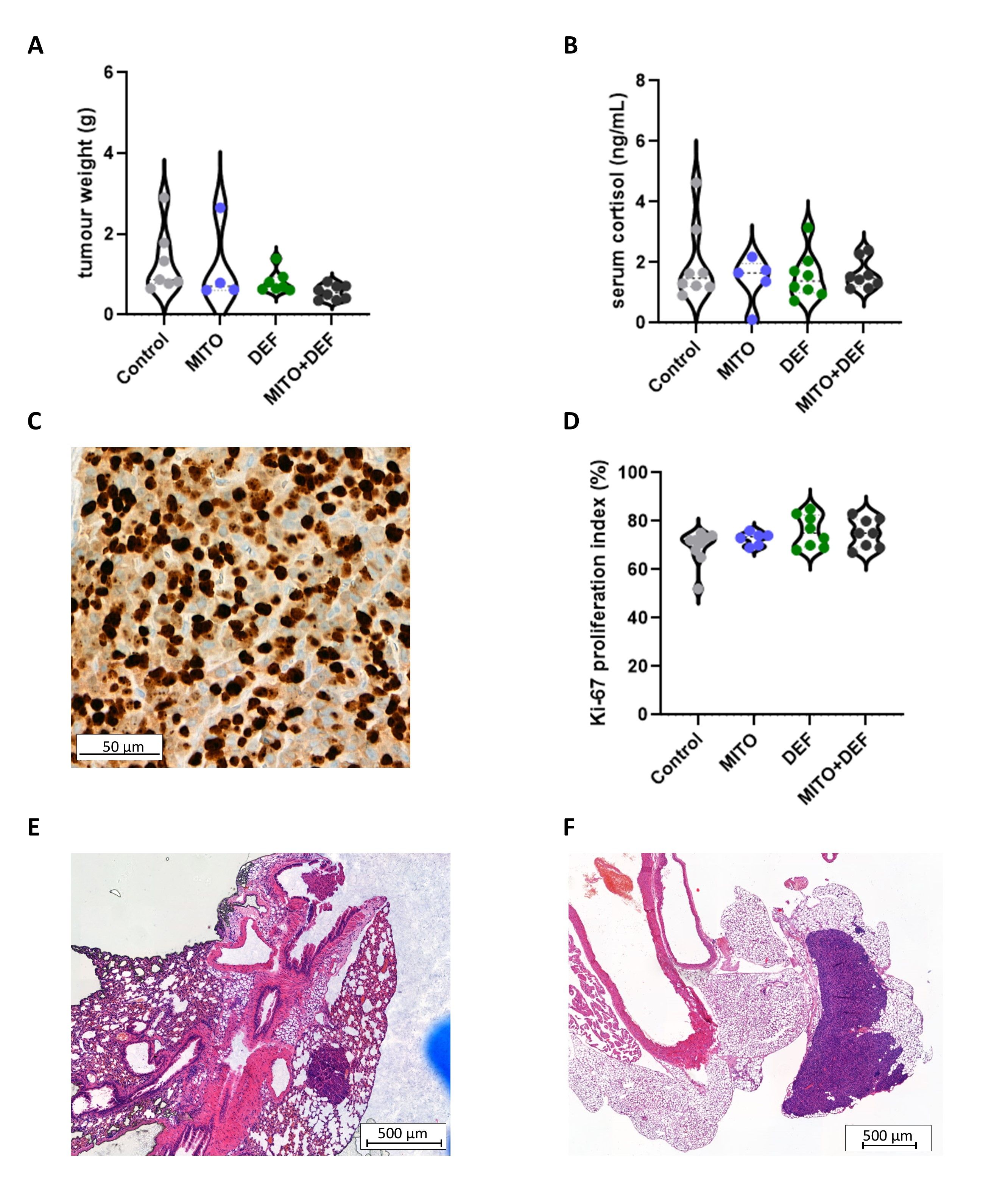

Supplement: Supplementary file 1 [file ijms-26-06539-s001.zip › SF6_1000.jpg]
